# A Rare Case of Polymerase Chain Reaction-Negative Severe Clostridioides difficile Infection

**DOI:** 10.7759/cureus.50403

**Published:** 2023-12-12

**Authors:** Charity C Iheagwara, Carlos Cantu Lopez, Onyinye N Otaluka, Byron Okwesili, Vadim Belinski, Ala Muhanna, Bereket Tewoldemedhin, Jihad Slim, Maria Szabela, Jack Boghossian, Yatinder Bains

**Affiliations:** 1 Infectious Diseases, Saint Michael's Medical Center, Newark, USA; 2 Internal Medicine, Howard University Hospital, Washington, USA; 3 Epidemiology and Public Health, George Washington University, Washington, USA; 4 Internal Medicine, Saint Michael's Medical Center, Newark, USA; 5 Internal Medicine, University of Nigeria, Enugu, NGA; 6 Gastroenterology and Hepatology, Saint Michael's Medical Center, Newark, USA; 7 Internal Medicine, Suburban Community Hospital (Lower Bucks Hospital), Bristol, USA; 8 Gastroenterology, Saint Michael's Medical Center, Newark, USA

**Keywords:** c. difficile colitis, pseudomembranous colitis, c. diff, cdi, diarrhea, clostridioides difficile infection

## Abstract

Accurately diagnosing *Clostridioides difficile* infection (CDI) is crucial for effective patient management. A misdiagnosis poses risks to patients, leads to treatment delays, and contributes to infection transmission in healthcare settings. While using polymerase chain reaction (PCR) to amplify the toxin B gene is a sensitive method for detecting toxigenic *C. difficile*, there is still a risk of false-negative results. These inaccuracies could have significant consequences for diagnosing and treating CDI, emphasizing the need for careful consideration and other diagnostic approaches. This case report highlights a patient with severe CDI who had negative PCR and toxin and a biopsy showing pseudomembranous colitis on further testing due to persistence and worsening of symptoms. In the diagnosis of *C. difficile* infection, healthcare providers should consider clinical symptoms, although diarrhea, which is a major sign of CDI, can be due to other causes. Even in the presence of negative PCR results, if a patient displays symptoms consistent with* C. difficile*-associated disease, healthcare providers may still contemplate treatment.

## Introduction

*Clostridioides difficile* infection (CDI) poses a significant healthcare challenge, and an accurate diagnosis is crucial for effective treatment. Polymerase chain reaction (PCR) tests have become a cornerstone in detecting *C. difficile*, offering high sensitivity. However, despite advancements in diagnostic techniques, the issue of false-negative PCR test results persists [1]. These false negatives pose a significant challenge to the prompt identification and management of CDI.

A study by Murad et al. [1] investigated the occurrence of false-negative results in *C. difficile* testing. The study underscores that even with sophisticated testing methodologies, false negative results can impact the diagnosis and subsequent treatment of CDI. This complexity arises from the intricate nature of the infection and the need for accurate diagnostic tools. In this context, understanding the factors contributing to false-negative PCR tests is essential. Challenges include variations in test sensitivity, the presence of non-toxigenic strains, and the potential for molecular assays to detect the organism without clinical relevance [2,3].

As the medical community grapples with these challenges, a comprehensive understanding of false-negative PCR results in CDI is imperative for refining diagnostic strategies and ensuring effective treatment. CDI is a major healthcare concern, with an increasing incidence, severity, morbidity, and mortality rate of 6-15% [2].

## Case presentation

A 71-year-old male with a medical history of gastroesophageal reflux disease (GERD), hyperlipidemia, benign prostatic hyperplasia (BPH), chronic obstructive pulmonary disease (COPD), and depression presented to the emergency room with complaints of recurrent diarrhea that had been present for two months. He noted having about eight to ten bowel movements daily. There was periumbilical pain and nocturnal diarrhea, but the diarrhea was not associated with meals. He denied any recent travel or change in diet. He acknowledged antibiotic use within two months of his presentation. He had a colonoscopy six months prior to the presentation, which was normal. He had repeated the EGD two months earlier due to the initial finding of a gastric ulcer on the initial EGD. He denied fever, chills, nausea, vomiting, melena, weight loss or hematochezia. He quit smoking 15 years earlier. He had no history of malignancy in his family.

On presentation to the emergency department, his vital signs were as follows: blood pressure was 95/60 mmHg, pulse rate of 100 per minute, respiratory rate of 18 per minute, temperature was 98.1 °F, and oxygen saturation was 93% on room air. Physical examination was significant for mild conjunctival pallor, dry tongue, and poor skin turgor. Intravenous fluids were administered as the patient was dehydrated with persistent diarrhea (Table 1).

**Table 1:** Baseline laboratory investigations on admission

Component	Results	Normal value	Units
WBC	30.60 × 10^3^	4.4–11 × 10^3^	U/L
Hemoglobin	13.5	13–17.7 g/dL	g/dl
Hematocrit	39.2	37.5–51	%
Platelets	257 × 10*3	150–450 × 10^3^	µL
Sodium	130	136–145	mmol/L
Potassium	5.3	3.5–5.3	mmol/L
Chloride	98	98–110	mmol/L
Bicarbonate	24.1	20–31	mmol/L
BUN	52.0	6–24	mg/dL
Creatinine	1.4	0.6–1.2	mg/dL
CRP	19.4	0.0–0.8	mg/dL
Glucose	144	70–140	mg/dL
AST	44	10–36	U/L
ALT	20	10–49	U/L
Alkaline phosphatase	76	40–116	U/L
Tissue transglutaminase (tTG-IgA)	<2	0–3	U/mL

Empiric oral vancomycin was started, and stool studies were requested, including PCR for *C. difficile* toxin. CRP and serum creatinine were elevated, the stool test for *C. difficile* PCR for toxin was negative, and empiric vancomycin was discontinued. The Clostridium molecular assay was subsequently negative. HIV was ruled out. Stool culture was negative for Salmonella, Shigella, Campylobacter, and *E. coli* O157:H7. There were no ova or parasites found in the stool. Tissue transglutaminase IgA and pancreatic fecal elastase were negative. A CT scan of the abdomen and pelvis with IV contrast revealed small ascites and pancolitis. The patient continued to have diarrhea and was upgraded to the critical care unit for hypovolemic shock within 24 hours of hospital admission, and he was requiring pressors and oxygen via nasal cannula.

**Figure 1:**
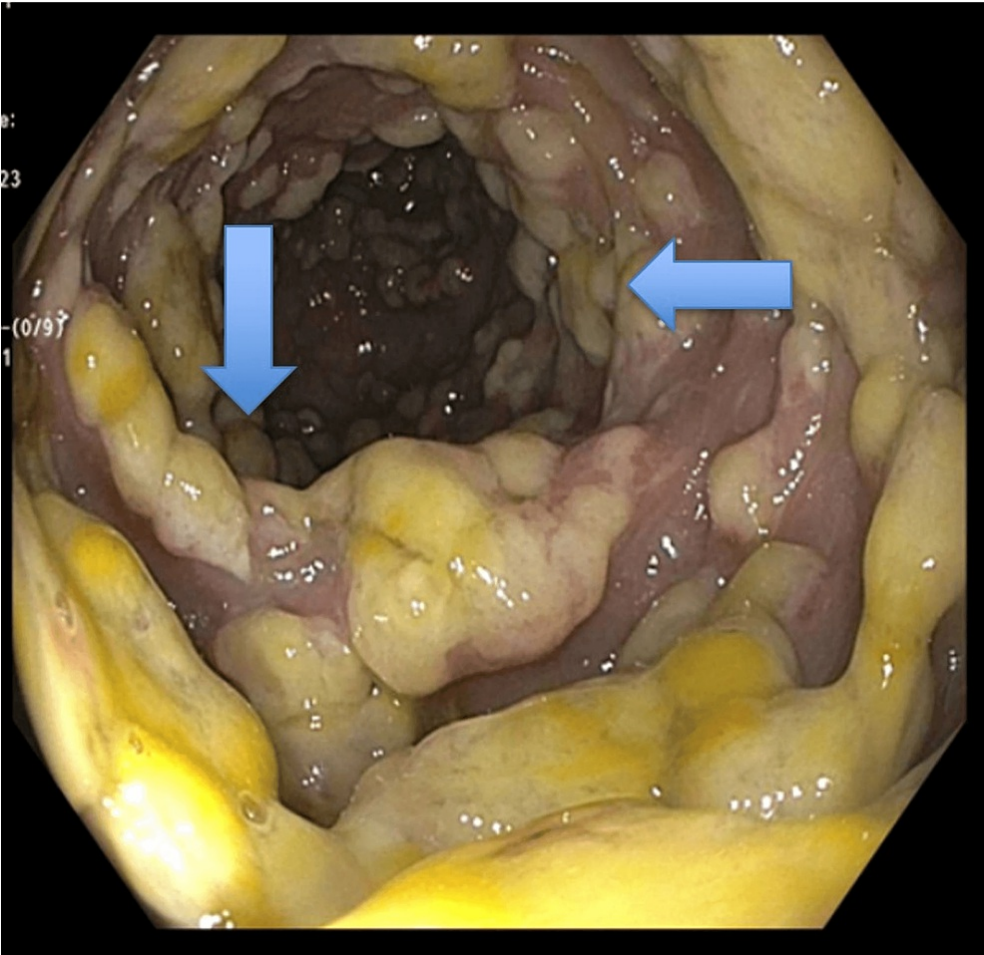
Colonic mucosa showing gross pathology consistent with pseudomembranous colitis (blue arrows).

**Figure 2:**
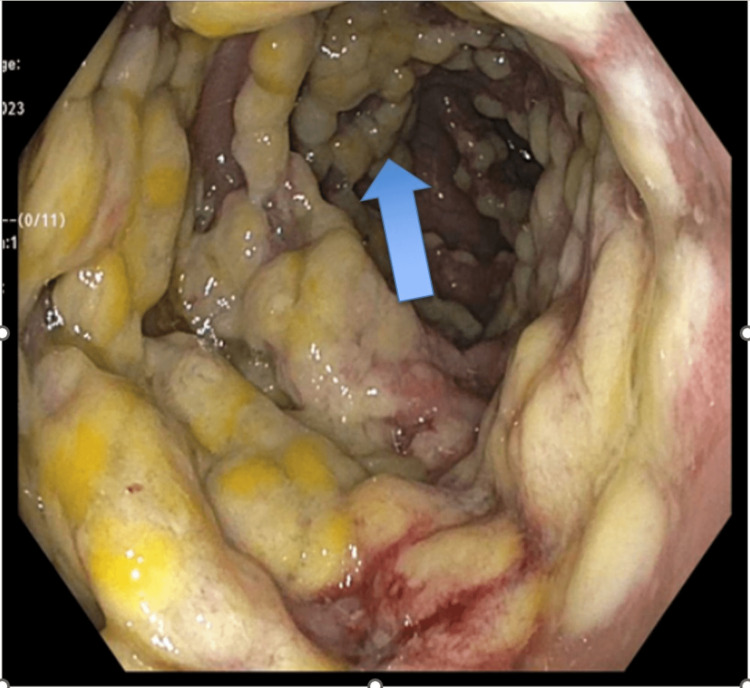
Colonic mucosa showing gross pathology consistent with pseudomembranous colitis (blue arrow).

**Figure 3:**
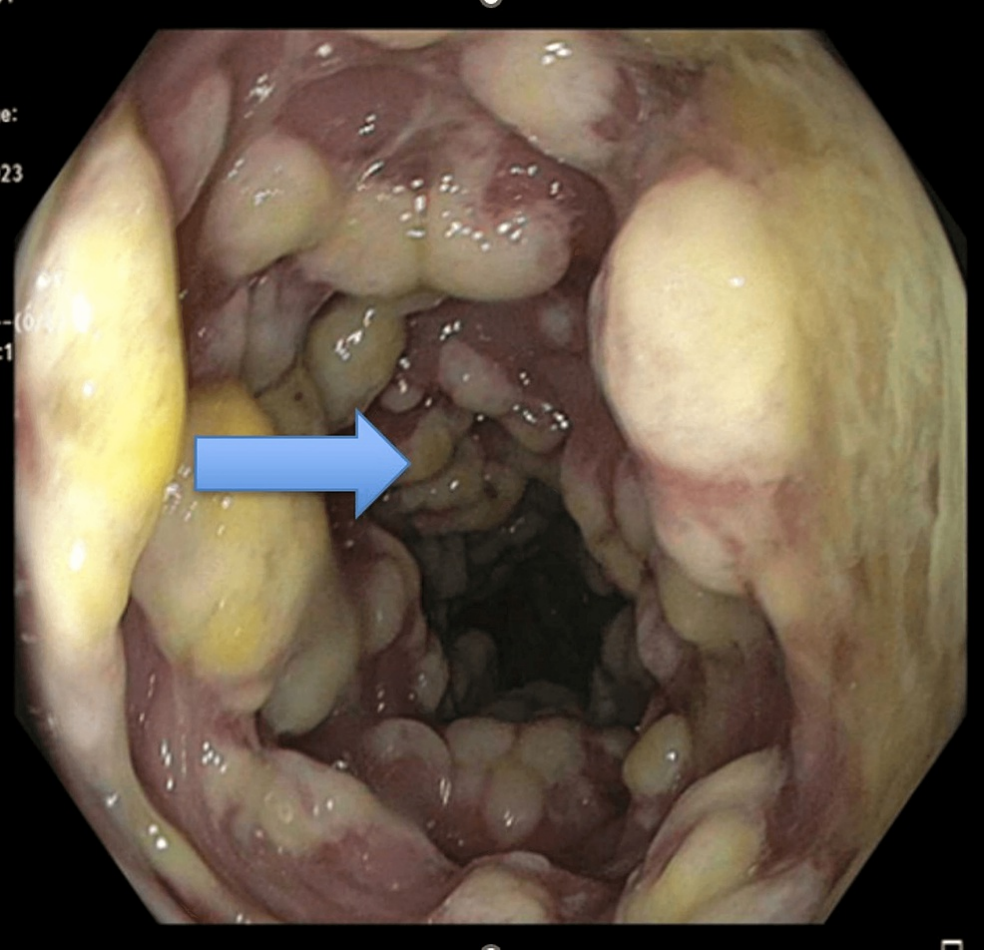
Colonic mucosa showing gross pathology consistent with pseudomembranous colitis (blue arrow).

**Figure 4:**
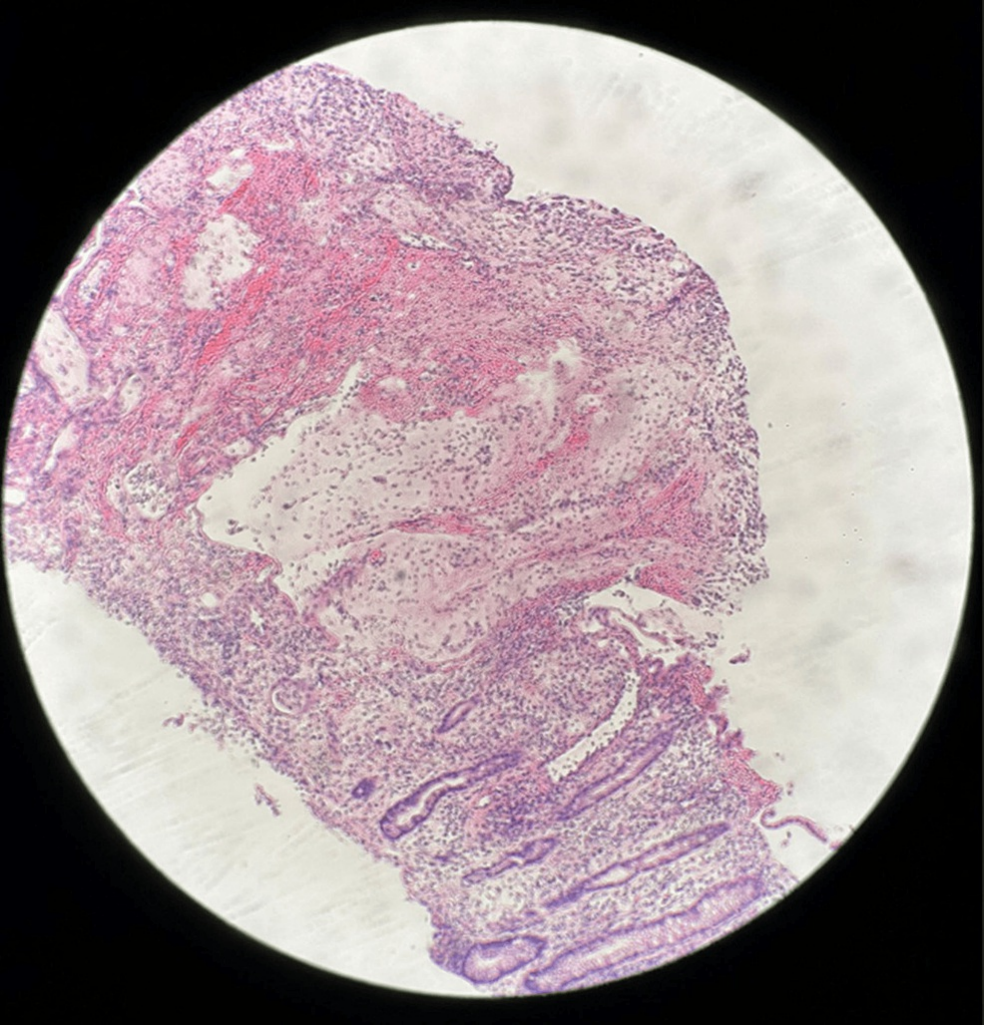
Benign polypoid colonic mucosa showing histology consistent with pseudomembranous colitis.

The colonoscopy and biopsy of the intestinal tissue showed benign polypoid colon mucosa with gross pathology consistent with pseudomembranous colitis (Figures 1-3) and histology consistent with pseudomembranous colitis (Figure 4). There was no definitive polyp, dysplasia, or malignancy noted via biopsy. The patient was treated with oral vancomycin with a marked clinical response. The diarrhea resolved, and the patient was discharged on oral vancomycin without any sequelae. His serum creatinine was 1.4 (normal range: 0.55-1.02 mg/dL) on admission and 0.8 mg/dL on the day of discharge.

The main reason for the negative PCR result lies mainly in the variation in the sensitivity of the molecular test, as noted in the case report. Since being discharged from the hospital, the patient visited the gastroenterology clinic once and denied any recurrence of diarrhea. Subsequent visits to his primary care doctor and other subspecialty clinics revealed no concern for diarrhea or other gastrointestinal symptoms.

## Discussion

The diagnosis of CDI involves identifying symptoms, typically watery diarrhea, along with positive results from an unformed stool test for *C. difficile* toxins. Alternatively, detection of toxigenic *C. difficile* or the presence of pseudomembranous colitis through colonoscopy or histopathologic findings is also indicative of CDI [4].

*C. difficile* colonization is defined as the presence of the bacteria without symptoms, increasing the risk for CDI if colonization is by toxigenic strains. CDI is defined as the presence of three or more loose stools in less than 24 hours and a laboratory diagnosis [2,4]. Even though CDI is a major concern for the healthcare system worldwide, there is no consensus regarding the optimal laboratory test for confirmation of CDI.

Currently, we have different options for laboratory diagnosis, including the toxic culture, cell cytotoxicity assay, and nucleic acid amplification test with the highest sensitivity levels. And for the highest specificity levels, cell cytotoxicity levels [5]. The gold standard for CDI is stool toxigenic culture, a two-step technique that isolates the bacteria and then determines if the isolated strain is a toxin producer or not. The cell cytotoxicity assay serves as the gold standard for toxin detection, and it can be performed directly or as a two-step approach in a toxigenic culture. It has high sensitivity and specificity, 0.94-1.0 and 0.99, respectively. The main disadvantages of this test include its time to perform and its complexity, which requires special training to complete the task. Nucleic acid amplification testing detects in a single step if the isolated strain is a toxin producer. This test has a 90% sensitivity and specificity [6].

There is no clear consensus on a definitive diagnostic method for *C. difficile* infections, and multiple approaches can be used for the laboratory diagnosis of CDI [7]. Different two- or three-step algorithms can be followed, considering a positive first test with high sensitivity and one confirmatory test with high specificity [7-10]. This is a cost-effective approach as well.

Diagnostic laboratory methods can be categorized into three groups based on their aim. Culture-based methods include toxicity testing. Methods focused on detecting strains that produce toxins include cell cytotoxicity assays and molecular methods aimed at detecting the toxin B gene, which include PCR testing [8]. Each method has different sensitivity and specificity, and combining these methods in a two- or three-step approach increases the opportunity for a prompt and proper diagnosis [7-10]. Rapid assays have a low sensitivity of 30-50% and should be accompanied by molecular tests, which have a specificity of >90% [4].

In the analysis of 432 samples of stool from symptomatic patients tested by glutamate dehydrogenase (GDH) assay, enzyme immunoassay (EIA) for toxin A and B, cell culture cytotoxicity neutralization (CCCN) assay, and PCR, the following observations were made when the results were compared with toxigenic culture [11]: the GDH assay demonstrated a sensitivity of 83.1%, a specificity of 96.7%, a PPV of 83.1%, and an NPV of 96.1%. An algorithm was used between the GDH assay and the EIA (plus the cell cytotoxicity assay if the EIA was negative). EIA had a sensitivity of 58.3%, a specificity of 94.7%, a PPV of 68.9%, and an NPV of 91.9%. PCR: sensitivity 94.4%, specificity 96.3%, PPV 84%, NPV 98.8%.

PCR only had the highest sensitivity and NPV compared to other methods (which include a combination of GDH and EIA and a combination of GDH and PCR), which makes this single study the best for an accurate diagnosis of CDI. Although surveillance has shown an increase in the diagnosis of CDI with NAAT (PCR) compared to toxin enzyme immunoassays, these tests may be falsely negative [12].

In certain situations, antibiotics may be started empirically as a precautionary measure while awaiting laboratory confirmation, considering gastrointestinal symptoms and the severity of *C. difficile* infections. In such cases, treatment may not be ruled out solely based on negative PCR results. Contact isolation precautions should be taken pending CDI test results per IDSA and may be continued for at least 48 hours after the resolution of the diarrhea [4]. IDSA also recommends the initiation of empiric antibiotic therapy pending laboratory confirmation of CDI or in cases of fulminant CDI [4]. An overall decision should consider clinical presentation and laboratory work-up.

Actual recommendations from the Infectious Disease Society of America (IDSA) suggest the use of stool toxin as a part of a multistep algorithm rather than NAAT by itself, especially in institutions where there are no criteria for stool submission [4]. In institutions where there are criteria for stool submission and patients with clinical symptoms suggesting CDI, the use of NAAT (PCR) alone or a multistep is the most sensitive method compared to a toxin test alone, per the IDSA [4].

## Conclusions

PCR tests for *C. difficile* toxin can be falsely negative and may pose a delay in the prompt diagnosis and treatment of *C. difficile* colitis. Healthcare providers must employ a blend of clinical judgment, PCR outcomes, and other diagnostic approaches to make well-informed decisions concerning the treatment of *C. difficile*, as this will ensure optimal management of the patient and decrease morbidity and mortality associated with the condition. It will also aid with the reduction in transmission of *C. difficile* infection among healthcare workers and across patients, as appropriate contact isolation is required to reduce spread.

Prompt diagnosis and treatment would impact the healthcare system by reducing morbidity and mortality. It is important to consider risk factors, clinical presentation, and laboratory assays to decide on optimal treatment, taking into account multiple factors for a better prognosis. Laboratory assays should always be accompanied by clinical interpretation.
